# Effect of High-Intensity Interval Training and Intermittent Fasting on Body Composition and Physical Performance in Active Women

**DOI:** 10.3390/ijerph18126431

**Published:** 2021-06-14

**Authors:** Alejandro Martínez-Rodríguez, Jacobo A. Rubio-Arias, José M. García-De Frutos, Manuel Vicente-Martínez, Thomas P. Gunnarsson

**Affiliations:** 1 Department of Analytical Chemistry, Nutrition and Food Sciences, Faculty of Sciences, University of Alicante, 03690 Alicante, Spain; 2 Alicante Institute for Health and Biomedical Research (ISABIAL Foundation), 03010 Alicante, Spain; 3 Department of Education, Faculty of Education Sciences, University of Almería, 04120 Almería, Spain; jararias@ual.es; 4 Faculty of Sport, San Antonio Catholic University of Murcia, 30107 Murcia, Spain; jmgarcia887@ucam.edu; 5 Faculty of Health Sciences, Miguel de Cervantes European University, 47012 Valladolid, Spain; mvmartinez11006@alumnos.uemc.es; 6 Department of Nutrition, Exercise and Sports, University of Copenhagen, 2100 Copenhagen Ø, Denmark; tgunnarsson@nexs.ku.dk

**Keywords:** nutrition, diet, exercise, health promotion

## Abstract

Nutritional strategies may have an effect on body composition and physical performance. Intermittent fasting (IF) is an eating pattern that cycles between periods of eating and fasting in specified time periods. Moreover, it is a common strategy among members of the athlete population that are looking for weight loss. However, this strategy may negatively affect physical performance, as compared to other weight loss strategies. The main purpose of this research was to use a cross-over design to study the effects of HIIT, with or without intermittent fasting, on muscular and anaerobic performance in 14 active women (27 ± 6 y). To assess performance, body composition (anthropometry), hand-grip strength, and counter-movement jump (CMJ) height was measured, and a 30 s Wingate test was completed assessed. HIIT + IF reduced fat mass (1 kg, *p* < 0.05, d = 1.1; 1.5%, *p* < 0.01, d = 1.0) and increased CMJ height (6.2 cm, *p* < 0.001, d = 1.8). In addition, the change in CMJ height in HIIT + IF was higher over HIIT (5.2 cm, *p* < 0.001, d = 1.9). In conclusion, intermittent fasting could be a nutritional strategy to decrease fat mass and increase jumping performance. However, longer duration programs would be necessary to determine whether other parameters of muscle performance could be positively affected by IF.

## 1. Introduction

Weight loss and improved body composition (decreased body fat and/or increased muscle mass) through physical activity and dietary modifications can help decrease the risk of overweight-related diseases [1]. Intermittent fasting (IF), i.e., a dietary strategy including periods of normal food and beverage consumption interspersed with periods of energy-intake suppression or fasting [2], is considered an alternative to daily caloric restriction, a broad term that encompasses a number of specific fasting protocols [3]. The aim is to create a net reduction in energy intake that makes it lower than energy expenditure, triggering adaptive cellular responses that improve glucose regulation, increasing resistance to stress [4]. In a recent systematic review, authors have demonstrated that during fasting, cells activate pathways that enhance intrinsic defenses against oxidative and metabolic stress and eliminate or repair damaged molecules, thus creating a state of negative energy balance and inducing weight loss [5].

These programs typically lead in energy modification or restriction, but these are not necessarily updated every day. In this sense, protocols of intermittent fasting can be grouped into three categories: alternate-day fasting, all-day fasting, and time-restricted eating [3]. Alternate-day fasting involves alternating between days of ad libitum feeding and fasting days, which usually consist of a single meal containing approximately 25% of daily caloric needs. Full-day fasting is perhaps the simplest form of intermittent fasting and usually consists of one or two days of complete fasting per week plus ad libitum feeding on the other days [6]. Time-restricted feeding involves following the same feeding routine each day, with a set number of hours designated as the fasting window and the remaining hours as the feeding window [7]. Thus, involving a training program is needed to validate this dietary strategy on its ability to preserve performance and study it changes on body composition.

High-intensity interval training (HIIT) is defined as repeated, intense efforts performed at an intensity eliciting >85% of maximal hear rate, interspersed with periods of recovery from low- or moderate-intensity exercise (active recovery) or complete rest [2,8,9]. The duration can be from 10 s to 6 min and is performed with a work-to-rest ratio of 1:1–3:1 [9]. In this sense, HIIT has been shown to be a time-efficient strategy to promote a number of beneficial physiological adaptations, including enhanced maximum oxygen consumption (VO2max) [10,11], increased fat oxidation [12,13], improved exercise capacity [11], increased insulin sensitivity [14], and improvements in body composition [15] in adult women.

The effects of a period of HIIT with intermittent fasting based on time-restricted eating have never been investigated, and it is not known whether intermittent fasting affects the outcome of HIIT in regards to body composition and physical performance. In the last ten years, HIIT has been presented as a popular method and form of training even for sedentary or active people seeking its healthy effects on cardiorespiratory and metabolic levels [16,17,18]. Although the number of publications on this subject has increased, the regulation of HIIT has not yet been sufficiently studied [19] due to the number of variables that can be manipulated: intensity and duration of effort and pause, work to rest ratio, type of pause (active or passive), number and duration of the series, and duration and type of recovery between series. Therefore, the purpose of this study was to compare the body composition and physical performance effects of HIIT, with or without intermittent fasting intervention, in active women. We hypothesized that HIIT combined with IF might contribute to enhance body composition as well as physical performance in active women.

## 2. Materials and Methods

### 2.1. Study Design

A cross-over design was used to compare the effects of 2 × 8 weeks of high-intensity interval training, without (HIIT) or with (HIIT + IF) intermittent fasting, on body composition and performance. The two intervention phases were separated by more than two weeks during which participants did not carry out programmed activity, in order not to alter the experimental phase.

**Figure 1:**
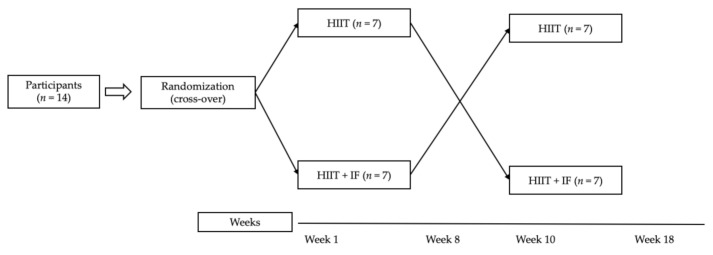
Flow diagram of cross-over study design.

A comparative and randomized cross-over experimental design was used to identify the effects of high-intensity interval training, without (HIIT) or with (HIIT + IF) intermittent fasting, on body composition and performance. All participants randomly performed two trainings (HIIT or HIIT + IF). In a first phase, participants were assigned either to the HIIT (*n* = 7) or HIIT + IF (*n* = 7) group. In the second phase, participants carried out the training that they had not performed in the first phase. The two intervention phases were separated by more than two weeks during which participants did not carry out programmed activity, in order not to alter the experimental phase (Figure 1).

### 2.2. Participants

Fourteen active, normal-weight women (age: 27 ± 6 years old, height: 167 ± 6 cm, weight: 58.9 ± 6.2 kg) participated in the study. Inclusion criteria were: (1) no muscular, ligamentous, bone, nerve, or joint pathology incompatible with the training program; (2) no present cardiovascular or cardiorespiratory problems; and (3) physically active in the last 5 years, according to the American College of Sports Medicine (ACSM) definition [20]. Members of the physically active population were considered to be those who perform physical exercises or general vigorous training at least 3 times a week on non-consecutive days. The exclusion criteria were: (1) following pharmacological treatment or supplement (including the ingestion of stimulants such as caffeine); and (2) performing other sports activities that may influence the study results during their participation in the study. All participants were assessed in the same menstrual cycle phase (late follicular, after the menstruation reported by the subjects) to avoid the effects of menstrual cycle phase on body composition and physical performance [21].

Ethics approval was granted by Ethics Committee of San Antonio Catholic University of Murcia (identifier: CE061811). All participants provided written informed consent prior to inclusion. All study procedures were conducted following the principles of the Declaration of Helsinki. The study was registered as a clinical trial in Clinicaltrial.gov (identifier: NCT04404413).

### 2.3. Body-Composition Assessment

Body-composition assessment was performed following standardized protocol [22], which included for subjects: basal assessment after 12 h overnight fasting, wearing minimal clothes (underwear), and participating in no exercise 24 h before the assessment. Anthropometric measurements were performed as body-composition assessment (fat mass, muscle mass, and residual mass). Restricted profile was performed by a Level 3 ISAK criterion anthropometrist following International Society for the Advancement of Kinanthropometry (ISAK) guidelines. Body mass and height measurements were performed using a digital scale with 0.1 kg accuracy (Tanita BC-545, Tokyo, Japan) and 0.1 cm accuracy stadiometer (SECA 213, Hamburg, Germany). Skinfolds, girths, and breadths were measured using professional anthropometric equipment (Smartmet, Jalisco, Mexico). Body-composition masses were estimated using validated equations. Carter’s equation was used to estimate fat mass [23], Lee’s equation to estimate muscle mass [24], and residual mass was obtained by difference.

### 2.4. Physical Performance Assessment

#### 2.4.1. Hand-Grip Strength

Hand-grip strength test was performed as an indicator of overall strength [25]. This test was conducted using a hydraulic hand-held dynamometer (Jamar, Preston, Jackson, Cape Girardeau County, MO, USA) with a 0.1 kg accuracy. During the test, subjects kept a standardized position (standing up, with the elbow in full extension) for 2–3 s of maximal pressure. All the subjects repeated the test twice with each hand, alternately. The researchers recorded the best score from the two attempts [26].

#### 2.4.2. Counter-Movement Jump

In the counter-movement jump (CMJ) test, the participants performed a maximum vertical jump starting from a standing position with arm swing not allowed. In addition, the subjects were required to flex their knees to a 90° angle. Moreover, to make sure the execution of the CMJ was correct, participants performed several familiarization trials before the testing session, and the protocol was standardized [27]. For the measurement of the CMJ [28], a contact platform was used (Chronojump Boscosystem, Barcelona, Spain) whose reliability and validity has been demonstrated [29]. Flight time was used to calculate the jump height. Participants performed three trials with 30 s of recovery period between them. The best jump was used for the subsequent analysis [30,31].

#### 2.4.3. Wingate Anaerobic 30 s Cycling Test

Subjects performed a Wingate Anaerobic 30 s cycling test with a validated cycloergometer (Monark 894E, Vansbro, Sweden). A 1-day (24 h) interval was required between the test and the training. Subjects were instructed to take these three tests at the same time of the day. Subjects were given a 5-min standard warm-up prior to the Wingate test [32], including two 3–5 min all-out sprints and a constant warm-up at 1 W/kg BM. Following a 5-min warm-up, the Wingate test was conducted according to the standard method of Bar–Or [33]. Peak power (PP) and mean power (MP) of the test were calculated via Monark Anaerobic Testing software (Version: 3.3.0.0). For statistics and results, relative MP (RMP) and relative PP (RPP) were estimated (W/Kg of subject body mass).

### 2.5. Nutrition Program: Intermittent Fasting

All individuals in both groups followed the same dietary/nutritional intermittent fasting program (intervention) and calorie intake adapted to their energy requirements. The overall balance of the diet was isocaloric, and no energy restriction was initially prescribed.

Intermittent fasting intervention consisted of a time-restricted feeding done every other day. Participants were instructed to not eat in <14 h of the day before, and consume breakfast as soon as possible after waking and to continue to eat following the diet intervals throughout the remainder of the day. The goal of the intermittent fasting based on time-restricted feeding was to promote fat loss while still providing adequate nutritional support for muscular and performance adaptations.

During the control period, the subjects had to follow their usual dietary intake based on a Mediterranean diet. However, during the intermittent fasting intervention, the participants restricted their habitual diet schedule for three alternate days one week and four alternate days the other week. The rest of the days, participants’ dietary intakes were the same as they were at the control period of the study without changes to caloric intake or distribution of meals. Dietary intake during the intervention periods were recorded, including the meal distribution schedule. All subjects kept their dietary, lifestyle, and training habits before the intervention program.

Energy intake was assessed using a 7-day food record, including cooking techniques and meal distribution. Diet information was analyzed using Dietsource software 3.0 (Novartis, Barcelona, Spain). Prior to the study, subjects received written information regarding food intake and timing. In addition, participants received a lesson in nutritional education before starting the nutrition program in order to be able to follow the intermittent fasting program during the intervention period without problems. During the intervention periods, subjects could contact the dietician at any time to resolve any doubts about the dietary programs. In both periods, distribution of macronutrients was the same. Protein ingestion was 1.8–2 g/kg of body mass [34,35,36], carbohydrate ingestion was 5–8 g/kg of body mass [34,35,36], and lipid intake was 1–1.2 g/kg of body mass [37].

### 2.6. Training Program

The HIIT intervention lasted for 2 × 8 weeks [38,39,40] and was interspersed by more than 2 weeks without training. In all sessions, subjects chose their own exercise intensity in response to a “maximal interval and session effort” [41]. The subjects performed the training three times per week, with the training sessions separated by 48 h [42,43,44]. Training sessions were supervised by a sports science graduate to ensure that the purpose of each training session was fulfilled in accordance to the interval and session effort. HIIT sessions lasted 40 min and were similar throughout the intervention period. The training session consisted of 3 × 10 repetitions of 30 s of aerobic exercises all out (cycling, rowing, and running) interspersed by 30 s of rest (passive recovery) [43]. The first set was performed on a cycle ergometer, (Technogym Bike Med Technogym SpA, Cesena, Italy) the second on a rowing ergometer (Model D, Concept 2, Morrisville, VT, USA), and the third on a motorized treadmill (Excite Run 1000 Med,Technogym SpA, Cesena, Italy).

### 2.7. Statistical Analyses

Statistical analysis was performed with the Statistical Package for the Social Sciences (SPSS, version 21, SPSS Inc., Chicago, IL, USA) in the Windows environment. Standard descriptive statistics were performed (mean and SDs). Descriptive data are presented as mean ± SD. For inferential analysis, a Shapiro–Wilk test was performed to establish the normality of sampling distribution. In addition, two-way analysis of covariance (ANOVA) with repeated measures was conducted to determine the main effects of time (training; pre- vs. post- intervention) and the two types of intervention (group; HIIT and HIIT + IF) as well as interaction of time and training. Effect size (ES) was calculated using partial eta-squared (ηp^2^). Threshold values for ES were ≥0.01 (small), ≥0.06 (moderate), ≥0.14 (large), and ≥2.0 (very large) [45]. Moreover, a Bonferroni post-hoc test was performed to analyse the effects of time on each type of training (HIIT and HIIT + IF). Additionally, Cohen’s d for repeated measures was estimated, threshold values for Cohen’s d ES were ≥0.1 (small), ≥0.3 (moderate), ≥1.2 (large) and ≥2.0 (very large) [46].

## 3. Results

Nutritional assessment of energy intake reported a caloric restriction (10–20% reduction in weekly energy intake) during the IF period. This restriction was not noticed by the athletes, who no reported feelings of having a calorie restriction in their diet.

Table 1 provides the summary statistics for anthropometry variables. A significant main effect for the training*group was observed in triceps (F = 7.407; *p* = 0.017), biceps (F = 4.937; *p* = 0.045), and leg skinfolds (F = 15.369; *p* = 0.002) and main group effect in waist (F = 11.220; *p* = 0.005).

**Figure 2:**
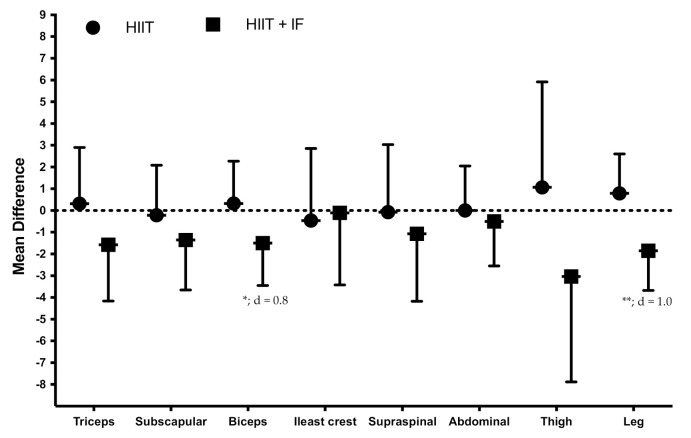
Effects of training on skinfolds (mm; mean difference post-training vs. pre-training: mean ± standard deviation). d, effect size; significant differences between pre- and post-training, * = *p* < 0.05; ** = *p* < 0.01.

**Figure 3:**
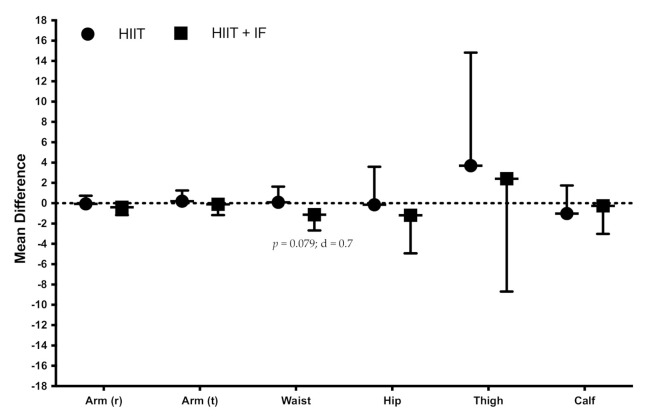
Effects of training on girths (cm; mean difference post-training vs. pre-training: mean ± standard deviation). r, relaxed; t, tensed; d, effect size.

However, no significant differences in the intervention-induced changes were observed between HIIT and HIIT + IF on skinfolds (Figure 2) and girths (Figure 3).

Concerning the body composition results, the significant main effect in the training * group was observed on fat mass (%, F = 14.084, *p* = 0.002; kg, F = 26.421, *p* < 0.001) (Table 2).

**Figure 4:**
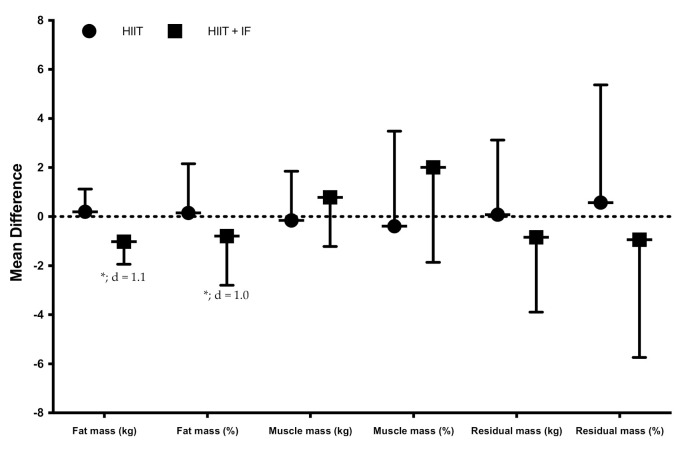
Effects of training on body composition (mean difference post-training vs. pre-training: mean ± standard deviation). d, effect size, significant differences between pre- and post-training, * = *p* < 0.05.

Additionally, the analysis of the pair comparison showed a significant decrease in the fat mass of the HIIT + IF group (%, *p* = 0.006, d = 1.0; kg, *p* = 0.003, d = 1.1) (Figure 4). However, no differences were observed between groups.

Finally, significant interactions were observed in the strength of the left arm (training effects) and height of the jump CMJ (training, group and training*group) (Table 3).

**Figure 5:**
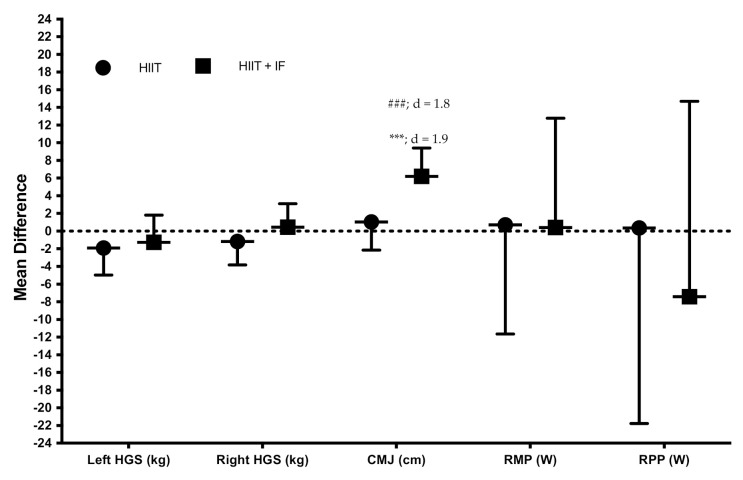
Effects of training on performance (mean difference post-training vs. pre-training: mean ± standard deviation). d, effect size, *** = *p* < 0.001 significant differences between pre- and post-training; ### = *p* < 0.001 significant differences between groups after training.

The HIIT + IF group showed a significant increase in CMJ jump height (35.43%, *p* < 0.001, d = 1.8). In addition, significant differences were observed between groups after training on CMJ (*p* < 0.001; d = 1.9) in favour of the HIIT + IF group and power in favour of the HIIT + IF group. On the other hand, differences were observed between groups before and after the training on RMP (W) (see Figure 5).

## 4. Discussion

The aim of the present analysis was to compare the body composition and physical performance effects of HIIT or HIIT and IF in active women. Initially, authors hypothesized that HIIT and IF could improve their body-composition variables as well as physical performance. The main finding of the study was that a HIIT + IF training program lead to a significant decrease in fat mass and an increase in jumping performance. However, when the HIIT training program was not combined with IF, it did not induce a decrease in fat mass or improvements in jump height.

Taken together, our results suggest a moderate change in fat mass and its related variables, such as triceps, biceps and leg skinfolds, without significant differences post-intervention. The women participating in the study maintained the same caloric intake in both interventions. The only variation was the timing of the intakes where the HIIT + IF group performed intermittent fasting, for which the participants developed a decreased energy intake by themselves (10–20% of total energy intake).

Fasting strategies, with a greater compatibility with the human circadian rhythm that leads to positive effects on cardiometabolic health [47], have been shown to also be useful, on the one hand, in reducing glucose oxidation and, on the other hand, by increasing fatty-acid oxidation [48]. Therefore, this state of metabolic flexibility could have a greater impact on the reduction of fat mass in individuals. In this sense, studies with fasting interventions and without caloric restriction have observed significant differences in the decrease of fat mass (2–3%) in the study population [49]. In our case, the results of the present study show a reduction in the percentage of body fat at the end of HIIT and HIIT + IF intervention of −0.3% and 1.0%, respectively.

Although no significant changes were observed in these subcutaneous fat variables (skinfolds), the HIIT + IF group did show a significant decrease in waist circumference related to abdominal obesity [50]. The results, which obtained improved waist perimeter (reduction), are in line with other studies that show that intermittent fasting effectively and favorably altered cardiovascular variables [51], in this case, because of a decrease in the visceral adipose tissue.

Regarding time and the gender influence on the change in body composition with the same training, this may be explained because, as shown in other studies [52], regulating the circadian rhythm has a positive effect on body weight and energy metabolism in women.

In addition, as it has been presented in numerous epidemiological studies, waist circumference is associated with cardiometabolic risks and pathologies, which extensively negatively affect public health [53,54]. The absence of significant changes could be due to the fact that the interventions in which a significant reduction in fat mass was observed were of longer duration (>12 weeks) than those used in the present study (8 weeks) [49,55]. Although scientific publications present evidence of changes in body composition regarding fasting as a nutritional strategy in less than 8 weeks for elite male athletes (4-weeeks) [56], an 8-week duration does not appear to be a sufficient stimulus to promote metabolic flexibility in our study population (active women). Indeed, 4 weeks for active males are not enough to improve the body composition [57], but 6 weeks was enough to find positive changes in women over 60 years [58].

Thus, it appears that changes in body composition are related to both the time of application of the fasting strategy and the population in which it is applied. Regarding performance adaptations, two aspects were found to be highlighted. Although there were no significant increases in muscle mass in the HIIT + IF group, the results showed a significant decrease of the leg skinfold. Likewise, these women compared to those in the HIIT group also reported significant improvements in jumping performance.

These adaptations could have occurred due to the intermittent fasting: the pattern of intakes throughout the day were more concentrated in the daytime phase of the study. Intakes were close to training; therefore, these intakes seem to have provided more energy and nutrients both before and after training. This can be interpreted as meaning that, in HIIT with IF, intakes in sensitive phases close to training are more important [59] and that they can favor, to a greater extent, adaptations to HIIT and, consequently, an improvement of the CMJ in the participants who followed an HIIT + IF regimen.

It is important, however, to consider the current limitations of the study. Although postprandial responses and hormonal adaptations are based on circadian biorhythm, different styles of energy balance might affect and have more impact on the human metabolism. For that reason, in further research, it could be interesting to include an extra group in which to develop an IF with an energy restriction, which would probably have a greater effect on body-composition variables, but it will be interesting to study what happens with athlete performance in different variables. In addition, a hormonal study control could be necessary to study muscle and performance adaptations to the different interventions.

## 5. Conclusions

In conclusion, intermittent fasting might be considered an alternative feeding schedule regimen for active women to significantly decrease fat mass and increase jumping performance. Additionally, nutritional strategies must be supervised by professional nutritionist.

## Figures and Tables

**Table 1:** Changes in anthropometric variables.

						ANOVA Main Effects								
						Training Effects			Group Effects			Main Effects (Training x Group)		
Variables	Group	Pre	SD	Post	SD	F	*p*	η^2^ p	F	*p*	η^2^ p	F	*p*	η^2^ p
Body mass (kg)	HIIT	58.9	6.2	59	5.8	1.099	0.314	0.078	0.814	0.383	0.059	1.802	0.202	0.122
	HIIT + IF	59.9	5.7	58.8	6.4									
**Skinfolds (mm)**	**Factor**	**Pre**	**SD**	**Post**	**SD**	**F**	***p***	**η^2^ p**	**F**	***p***	**η^2^ p**	**F**	***p***	**η^2^ p**
Triceps	HIIT	16.5	5	16.9	3.3	1.099	0.314	0.078	0.814	0.383	0.059	7.407	0.017	0.363
	HIIT + IF	17	4.8	15.4	3.9									
Subscapular	HIIT	10.3	2.4	10.1	1.7	1.099	0.314	0.078	0.814	0.383	0.059	2.177	0.164	0.143
	HIIT + IF	11.2	2.3	9.9	1.6									
Biceps	HIIT	5.1	2.3	5.5	2.4	3.402	0.088	0.207	1.968	0.215	0.116	4.937	0.045	0.275
	HIIT + IF	6.5	2.4	5.0	2.1									
Iliac crest	HIIT	13.5	3.9	13.1	3.8	0.256	0.621	0.019	1.849	0.197	0.125	0.068	0.798	0.005
	HIIT + IF	14.3	3.6	14.1	3.9									
Supraspinal	HIIT	10.9	4.7	10.8	3.5	0.825	0.380	0.060	0.066	0.801	0.005	0.852	0.373	0.062
	HIIT + IF	11.3	4.1	10.3	3.3									
Abdominal	HIIT	12.9	2.5	12.9	2.7	0.552	0.471	0.041	1.099	0.314	0.078	0.333	0.574	0.025
	HIIT + IF	13.6	3.4	13.1	3.1									
Thigh	HIIT	23.4	5.1	24.4	7.1	1.595	0.229	0.109	0.244	0.630	0.018	3.931	0.069	0.232
	HIIT + IF	25.7	7.1	22.6	4.3									
Leg	HIIT	16.4	4.7	17.2	4.9	2.336	0.155	0.152	0.412	0.532	0.031	15.369	0.002	0.542
	HIIT + IF	18.1	6	16.2	6									
**Girths (cm)**	**Factor**	**Pre**	**SD**	**Post**	**SD**	**F**	***p***	**η^2^ p**	**F**	***p***	**η^2^ p**	**F**	***p***	**η^2^ p**
Arm (relaxed)	HIIT	26.4	2.2	26.4	1.8	2.210	0.161	0.145	0.356	0.561	0.027	1.273	0.28	0.089
	HIIT + IF	26.7	2.1	26.3	2.1									
Arm (flexed and tensed)	HIIT	26.6	1.9	26.5	1.9	0.095	0.763	0.007	0.295	0.596	0.022	0.470	0.505	0.035
	HIIT + IF	26.3	1.9	26.2	1.9									
Waist	HIIT	68.5	3.8	68.6	3.8	3.954	0.068	0.233	11.220	0.005	0.463	3.382	0.089	0.206
	HIIT + IF	68.2	3.7	67.1	3.5									
Hip	HIIT	95.9	4.4	95.8	7.5	0.723	0.411	0.053	0.204	0.659	0.015	0.684	0.423	0.05
	HIIT + IF	96.9	6.4	95.7	6									
Thigh	HIIT	44.2	8.6	48.0	5.9	2.409	0.145	0.156	3.105	0.102	0.193	0.083	0.778	0.006
	HIIT + IF	48.4	5.9	50.8	12.5									
Calf	HIIT	36.21	2.31	35.21	4.996	1.409	0.256	0.098	0.010	0.923	0.001	0.534	0.478	0.039
	HIIT + IF	35.89	2.5	35.63	2.95									

Pre, pre-intervention; Post, post-intervention; SD, standard deviation; HIIT, high-intensity interval training; IF, intermittent fasting.

**Table 2:** Changes in body composition.

						ANOVA Main Effects								
						Training Effects			Group Effects			Main Effects (Time x Group)		
Variable	Group	Pre	SD	Post	SD	F	*p*	η^2^ p	F	*p*	η^2^ p	F	*p*	η^2^ p
Fat mass (kg)	HIIT	10.4	2.4	10.6	2.41	3.492	0.084	0.212	0.976	0.341	0.07	26.421	<0.001	0.67
	HIIT + IF	11.2	2.7	10.2	2.6									
Fat mass (%)	HIIT	17.6	2.9	17.9	2.8	3.466	0.085	0.211	0.535	0.477	0.044	14.084	0.002	0.52
	HIIT + IF	18.6	3.3	17.1	2.8									
Muscle mass (kg)	HIIT	21.0	2.2	20.81	2.83	0.684	0.423	0.05	1.797	0.203	0.121	1.632	0.224	0.112
	HIIT + IF	21.0	1.9	21.8	2.7									
Muscle mass (%)	HIIT	35.7	2.5	35.3	3.5	1.083	0.317	0.077	0.994	0.337	0.071	3.111	0.101	0.193
	HIIT + IF	35.2	2.9	37.2	4.9									
Residual mass (kg)	HIIT	18.1	2.9	18.17	2.1	0.392	0.542	0.029	0.241	0.631	0.018	0.737	0.406	0.054
	HIIT + IF	18.3	3.4	17.4	3.7									
Residual mass (%)	HIIT	30.5	3.5	31.0	3.3	0.032	0.86	0.003	0.687	0.424	0.054	0.906	0.36	0.07
	HIIT + IF	30.5	4.4	29.5	5.3									

Pre, pre-intervention; Post, post-intervention; SD, standard deviation. Fat mass: Carter’s equation; Muscle mass: Lee’s equation; Residual mass: by difference. HIIT, high-intensity interval training; IF, intermittent fasting.

**Table 3:** Changes in muscular and anaerobic performance.

						ANOVA Main Effects								
						Training Effects			Group Effects			Main Effects (Training x Group)		
	Group	Pre	SD	Post	SD	F	*p*	η^2^ p	F	*p*	η^2^ p	F	*p*	η^2^ p
Left HGS (kg)	HIIT	29.3	3.9	27.4	3.4	6.626	0.023	0.338	0.769	0.369	0.056	0.326	0.578	0.024
	HIIT + IF	29.4	3.8	28.1	3.0									
Right HGS (kg)	HIIT	29.6	4.2	28.4	3.3	0.435	0.521	0.032	1.266	0.281	0.089	3.265	0.094	0.201
	HIIT + IF	29.2	4.1	29.7	3.0									
CMJ (cm)	HIIT	17.5	4.3	18.5	3.9	30.21	<0.001	0.699	21.15	<0.001	0.662	22.46	<0.001	0.633
	HIIT + IF	17.5	4.2	23.7	4.4									
RMP (W)	HIIT	148.4	27.9	149.1	28.5	0.07	0.795	0.005	13.000	0.003	0.5	0.003	0.955	0.000
	HIIT + IF	134.6	30.9	135.0	20.9									
RPP (W)	HIIT	295.3	37.8	295.6	40.7	0.761	0.399	0.055	0.036	0.852	0.003	0.817	0.382	0.059
	HIIT + IF	297.0	42.1	289.6	46.8									

Pre, pre-intervention; Post, post-intervention; CMJ, counter-movement jump; SD, standard deviation; HGS, hand-grip strength; RMP, relative mean power; RPP, relative peak power; HIIT, high-intensity interval training; IF, intermittent fasting.

## Data Availability

The data presented in this study are available on request from the corresponding author. The data are not publicly available due to privacy restrictions.
